# Selenium-Enriched Foods Are More Effective at Increasing Glutathione Peroxidase (GPx) Activity Compared with Selenomethionine: A Meta-Analysis

**DOI:** 10.3390/nu6104002

**Published:** 2014-09-29

**Authors:** Emma N. Bermingham, John E. Hesketh, Bruce R. Sinclair, John P. Koolaard, Nicole C. Roy

**Affiliations:** 1 Food Nutrition & Health, Food & Bio-based Products, AgResearch Grasslands, Private Bag 11008, Tennent Drive, Palmerston North 4442, New Zealand; E-Mails: bruce.sinclair@agresearch.co.nz (B.R.S.); nicole.roy@agresearch.co.nz (N.C.R.); 2 Institute for Cell & Molecular Biosciences, University of Newcastle upon Tyne, Newcastle NE2 4HH, UK; E-Mail: j.e.hesketh@newcastle.ac.uk; 3 Bioinformatics & Statistics AgResearch Grasslands, Private Bag 11008, Tennent Drive, Palmerston North 4442, New Zealand; E-Mail: john.koolaard@agresearch.co.nz; 4 Riddet Institute, Massey University, Palmerston North 4442, New Zealand; 5 Gravida: National Centre for Growth and Development, the University of Auckland, Auckland 1142, New Zealand

**Keywords:** dietary selenium supplementation, glutathione peroxidase activity, meta-analysis, selenium, epigenetic regulation

## Abstract

Selenium may play a beneficial role in multi-factorial illnesses with genetic and environmental linkages via epigenetic regulation in part via glutathione peroxidase (GPx) activity. A meta-analysis was undertaken to quantify the effects of dietary selenium supplementation on the activity of overall GPx activity in different tissues and animal species and to compare the effectiveness of different forms of dietary selenium. GPx activity response was affected by both the dose and form of selenium (*p* < 0.001). There were differences between tissues on the effects of selenium supplementation on GPx activity (*p* < 0.001); however, there was no evidence in the data of differences between animal species (*p* = 0.95). The interactions between dose and tissue, animal species and form were significant (*p* < 0.001). Tissues particularly sensitive to changes in selenium supply include red blood cells, kidney and muscle. The meta-analysis identified that for animal species selenium-enriched foods were more effective than selenomethionine at increasing GPx activity.

## 1. Introduction

Selenium is an essential cofactor for approximately 25 selenoproteins [1,2], including the glutathione peroxidases (GPx1–8 [3]), selenoprotein P and thioredoxin reductases. GPxs are enzymes crucial for detoxification and protecting cells from oxidant damage. To date, eight isoforms of the GPx family have been identified; most of these forms are differentiated by their cellular (or tissue) location and substrates used and whether they incorporate cysteine rather than selenocysteine (Sec) [3,4]. The GPx family has been the focus of many reviews [3,4]. However, briefly, GPx1 (cGPx) is a cytosolic enzyme found in all tissues including blood cells; it reacts with damaging peroxides such as hydrogen peroxide. GPx2 (GSHPx-GI) and GPx3 (pGPx) also use peroxides as substrates but GPx2 is predominantly expressed in gastrointestinal cells whereas GPx3 is a secreted form found in plasma and milk [4,5]. GPx4 (PHGPx) is different in that it uses phospholipid hydroperoxides as substrates and is found in both mitochondria and cytosol; it is expressed in most tissues but is found in a high concentration in the testes. GPx4 has been proposed to have functions in apoptosis and protecting mitochondrial function from damaging radicals [6], sperm development [4] and embryonic development [7]. GPx1–4 all incorporate Sec [3]. GPx5 (eGPx) is found in the epididymis and has roles in fertility [3]. GPx6, also known as Olfactory metabolising protein (OMP), is only classed as a selenoprotein in humans, not in rodents [3], is located in the olfactory epithelium and has possible roles in olfaction [4]. More recently, GPx7 (NPGPx) has been described; like GPx5 it is a cysteine-based isoform, and is thought to prevent oxidative stress in breast cancer cell lines [4] and protein folding in the endoplasmic reticulum [3]. GPx8 was discovered using phylogenetic analyses [4] and also is thought to have a role in protein folding [3]. GPx5–8 are cysteine-based isoforms [3]. Selenium incorporation into selenoproteins (as SEC) occurs during their synthesis by a mechanism involving the 3′ untranslated region of mRNAs and trans-acting proteins [8].

Dietary deficiency of selenium has been shown to redistribute intracellular selenium among the selenoproteins and GPx proteins [9]. Synthesis of the various selenoproteins responds differently to changes in selenium supply [2] with GPx1 being highly sensitive and GPx2 and GPx4 more resistant to changes in selenium supply. In rats, dietary deficiency of selenium has been shown to redistribute intracellular selenium among several selenoproteins including GPxs [9]. There has been much interest in the effects of selenium supplementation on GPx activity (especially red blood cell GPx and plasma GPx3) and mRNA levels since these may provide potential biomarkers of selenium status [10,11,12,13].

Oxidant damage to DNA is a critical event in cancer development [14]. Evidence indicates that a polymorphism of the 3′ untranslated region of the GPx4 gene is functional [15] and associated with colon cancer risk [16]. This suggests that variants in GPx genes increase oxidant-induced DNA damage leading to increased cancer risk. Other studies have shown that, in addition to changes in the DNA sequence, there are changes occurring during cancer that are not in the gene sequence (epigenetic events). Alteration of methylation patterns in CpG islands (often located in DNA promoter) of some GPx genes have been shown in some cancers [17]. Research has shown that another type of epigenetic factor, microRNAs (miRNAs), is also important in human carcinogenesis [18]. These tiny molecules are short, single stranded non-coding RNAs that regulate gene expression by base pairing with target mRNAs at the 3′ untranslated region, leading to mRNA cleavage [19]. The 3′ untranslated region is critical for selenoprotein synthesis and therefore miRNAs (which target such regulatory regions) have the potential to play important roles in regulating GPx expression. In this regard, cell culture studies have recently indicated that changes in selenium supply cause altered expression of a number of miRNA, and altered expression of miR-185 was linked to regulation of GPx2 and Selenophosphate Synthetase 2 (SEPSH2) expression [20]. As a result, there is considerable interest both in the response of selenoprotein activity to selenium supplementation and in finding effective biomarkers of functional selenium status.

The effects of dietary selenium on different GPx activities are complex with differences between enzymes and tissues. Most authors have found an increase in GPx1 in response to dietary selenium in rodents [21,22,23] and selenoprotein expression, including GPx activity, in different tissues is affected by selenium availability to differing extents [24]. While some authors have assessed the effects of different selenium forms on GPx activities, and others have investigated the effects of selenium supplementation on GPx activity in different tissues [25], no one study has attempted to describe quantitatively the nature of the relationship between selenium supplementation and either the different GPx activities or the overall GPx activity (encompassing GPx1–4).

The human epigenome is vital to regulation of tissue functions [26]. The epigenome comprises interconnected, interdependent heritable processes that regulate gene expression (e.g., DNA methylation, histone modifications, non-coding RNAs). There is mounting evidence that these events have a major role in development and function of all cells and in the development of multi-factorial diseases in all stages of life; they allow plasticity of the phenotype in a fixed genotype [27]. Evidence suggests that specific nutrients can modulate epigenetic events [28,29], indicating it may be possible to reduce or even reverse the negative effects in multi-factorial illnesses by diet. Selenium is one nutrient which has been suggested to act through epigenetic mechanisms [30] either by affecting the availability of *S*-adenosyl methionine or the concentration of *S*-adenosyl homocysteine (inhibits of DNA methyltransferases) via the methionine-homocysteine cycle [31].

Epigenetic studies require a comprehensive understanding about the dose rate of the nutrient of interest, and the tissue in which to investigate changes in epigenetic regulation. For example, in the case of obesity, epigenetic mechanisms are largely specific to cell type [32]. In the present work, we used a meta-analysis approach to describe tissue differences in overall GPx activity in response to dietary selenium supplementation, and to investigate whether the degree of response varied according to animal species or form of dietary selenium supplementation. The meta-analysis approach, which is a widely used statistical tool to assess treatment effects across similar experiments conducted at different times, gives the advantage of reducing the risk of experimental bias arising from experimental conditions from single experiments. The meta-analysis described here provides essential information for designing appropriate studies in which to investigate the potential role of selenium in the epigenetic regulation of GPx activity.

## 2. Experimental Section

### 2.1. Database

This study was a meta-analysis of publications reporting the effects of selenium supplementation on GPx activity, and has been reported in accordance with the Preferred Reporting Items for Systematic reviews and Meta-Analyses (PRISMA) statement [33] with reference to the explanation and elaboration document [34]. Three of the authors (Nicole C. Roy, John E. Hesketh, Emma N. Bermingham) met in February 2011 to agree in advance on the protocol.

### 2.2. Information Sources and Searches

The primary author (Emma N. Bermingham) searched the scientific literature electronically in order to identify publications that estimated the effects of selenium supplementation on GPx activity. Online resources searched included OVID databases (Medline, BIOSIS, FSTA, CAB Abstracts), SCOPUS, and PUBMED. The search terms used to identify suitable publications included relevant terms covering selenium supplementation, gluthatione peroxidase, selenoprotein. We limited our database to investigate the effects of selenium supplementation on the activity of selenoproteins in healthy *in vivo* models. Our literature search was limited to publications reported in English, and those publications which reported selenoprotein activity in table format. Our initial investigations identified that GPx were the predominant selenoproteins investigated therefore a database was constructed using the publications that dealt only with dietary selenium supplementation and activity of GPx in tissues, plasma and red blood cells. The electronic searches commenced on 15 February 2011 and the last search was performed on 31 January 2012.

### 2.3. Eligibility Criteria

A “publication” was defined as a distinct piece of published work, be it a full paper or research communication (abstract) at a scientific meeting. Only publications in the English language were considered, but no date limits were set for inclusion. In all cases, data on selenium supplementation dose rates and methods and form (e.g., sodium selenite, selenomethionine), methodologies used to determine GPx activities, the animal species (including strain and generation if appropriate), the tissue studied, and the response of selenoprotein activity were recorded. Studies investigating alternative forms of selenium dosing—for example, injection, or dosage via water source—were not included in the analysis. Forms of selenium were classed either as basal diet (control diet), sodium selenite, sodium selenate, selenomethionine, selenium-enriched yeast (selenium-yeast; e.g., SelPlex) or selenium-enriched foods (e.g., mushrooms, animal tissue, onions, milk, *etc.*).

In order to compare animal species, all dietary supplementation data were normalised to mg/kg selenium for the dose of selenium given. The concentration of selenium in the diet was used as an indicator of selenium supplementation, rather than selenium intake, as few publications recorded intake data. Depletion and repletion studies were not included in the database. In cases where deficiencies of multiple nutrients were investigated, only the treatment groups relating to changes in selenium were used with adequate levels of the other nutrients.

The activity of GPx was standardised to a common unit (nmol NADPH oxidised/min/mg protein in the case of tissues, nmol NADPH oxidised/min/mL for plasma and nmol NADPH oxidised/min/mg haemoglobin for red blood cells). Literature in which the units of selenoprotein activity were not defined in the publication were not included in the database.

Within each publication, treatment groups were coded according to form, tissue and animal as illustrated in Table 1 where treatment groups are shown to be assigned according to the tissue studied. In addition, if appropriate, groups were also coded for length of dosing, as for example in the study of Toshiro *et al.* [35] in which chickens were fed two levels of selenomethionine for 35 days and tissue samples taken at day 35 of feeding.

**Table 1:** An example of coding treatments within a publication [35].

Experimental	Tissue	Length of Dietary	Level of	Selenium Form	Publication Treatment
Group		Treatment (Days)	Dose	(mg/kg Se)	Group
Se−	Erythrocyte	35	0	Selenomethionine	Ara_7
Se+	Erythrocyte	35	0.3	Selenomethionine	Ara_7
Se−	Kidney	35	0	Selenomethionine	Ara_10
Se+	Kidney	35	0.3	Selenomethionine	Ara_10
Se−	Liver	35	0	Selenomethionine	Ara_9
Se+	Liver	35	0.3	Selenomethionine	Ara_9
Se−	Muscle (pectoral)	35	0	Selenomethionine	Ara_11
Se+	Muscle (pectoral)	35	0.3	Selenomethionine	Ara_11
Se−	Muscle (femoral)	35	0	Selenomethionine	Ara_12
Se+	Muscle(femoral)	35	0.3	Selenomethionine	Ara_12
Se−	Plasma	35	0	Selenomethionine	Ara_8
Se+	Plasma	35	0.3	Selenomethionine	Ara_8

Se, selenium.

### 2.4. Study Selection and Data Collection Process

The primary author (Emma N. Bermingham) reviewed all publications identified from the electronic search, and assessed study eligibility in an unblinded, manner. A copy of all eligible publications was first obtained, either as a portable document format (PDF) file, or as a photocopy of the original paper document.

Two authors (Bruce R. Sinclair and Emma N. Bermingham) extracted relevant data from all eligible publications that were available. Data were entered into a computer spreadsheet (Excel version 2010, Microsoft, Redmond, USA).

### 2.5. Data Handling and Statistical Analysis

One author (John P. Koolaard) conducted all statistical analyses using computer software (Microsoft Excel 2010, and GenStat Version 14). We determined the effects of selenium form, tissue and animal species on the dose-response relationship between selenium supplementation and GPx activity. In each case, it was the slope of the dose-response relationship that was focussed on.

GPx activity and selenium dose were log (base 10) transformed, and thus the dose-response relationship was linearised. For selenium dose, a small constant of 0.01 was added before taking logs since some dose rates were zero. A weighted linear mixed model analysis using restricted maximum likelihood (REML) (GenStat Version 14) was used, where the weights for each observation were inversely proportional to the stated variance of the mean quoted in the publication. The publication number and coded treatment groups (within publication) were considered to be random effects in the model. The fixed effects were: the (log of) selenium dose, and its interaction with tissue, species and form, as well as the main effects of tissue, species and form group.

Classifications (e.g., animal, tissue, form groups) with less than 5 data points were not included in the meta-analysis.

## 3. Results

For the animal comparison, 40 publications were identified that met the selection criteria described in the Methods section (Table 2). Data from these were used here to determine the relationship between selenium dose and GPx activity. These publications were coded into 593 treatment groups (see Table S1).

### 3.1. Overall Relationship between Selenium Supplementation and GPx Activity

As indicated in Table 3, the meta-analysis included treatments groups in birds (59 treatment groups), horses (24 treatment groups), ruminants (14 treatment groups) and rodents (496 treatment groups). In animal species, liver was the predominant tissue investigated (160 treatment groups), followed by the gastrointestinal tract (68 treatment groups), plasma (60 treatment groups) and muscle (55 treatment groups). Sodium selenite (141 treatment groups), selenomethionine (99 treatment groups), and sodium selenate (85 treatment groups) were the predominate forms of selenium supplemented.

REML analysis of data across all the studies showed that the overall relationship between selenium supplementation and GPx activity (ignoring other factors) could be described by the equation: Log GPx activity = 1.822 (SE 0.146) + 0.645 (SE 0.116) × Log Dose. Both the form of selenium (*p* < 0.001) and tissue studied (*p* < 0.001) significantly influenced both the intercept and the slope of this relationship (Table 3), whereas animal species only influenced the slope (*p* = 0.03).

Quantitative analysis indicated that the selenium-enriched foods were more effective at increasing GPx activity compared to other forms of selenium including selenomethionine and selenium-yeast (indicated by the log Dose.Form in Table 3). As indicated by similar log Dose.Form values, sodium selenite, sodium selenate and selenomethionine decreased GPx activity. In addition, GPx activity in the red blood cells, kidney and muscle were least responsive (tissue × dose interaction as indicated by Log Dose.Tissue in Table 3) to selenium supplementation and GPx activity in the gastrointestinal tract most responsive.

**Table 2:** Description of the form of selenium studied for selenoprotein activity with changing concentrations of selenium in the diet for animal species and average gluthationine peroxidase (GPx) activity. The table indicates the number of treatment groups for each publication. A full description of the data set including methodology used can be found in Table S1.

Reference	Animal Species	*n* Treatment Groups	Diet Form	Tissue	Se Dose Rate (mg/kg)	GPx Activity (nmol NADPH ox/min/mg prot)
[23]	Rodent	5	Basal diet	Gastrointestinal tract	0.1	5850
			Selenium-enriched food		0.5; 1	6150–5950
			Selenium-yeast		1	8120
[36]	Rodent	45	Basal diet	Heart, kidney, liver, plasma, red blood cells	0.1	95–1023
			Sodium selenite		0.25; 0.5	125–1059
			Selenomethionine		0.25; 0.5	125–1063
			Selenium-yeast		0.25; 0.5	127–1011
[37]	Rodent	16	Basal diet	Plasma, red blood cells	0.025	54–476
			Sodium selenite		0.05; 1; 2	54–609
[38]	Ruminant	8	Basal diet	Muscle, red blood cells	0.16	14–80
			Sodium selenite		0.3	19–97
			Selenium-yeast		0.3	24–104
[39]	Rodent	4	Basal diet	Liver	0	71–77
			Sodium selenite		1	104–151
[40]	Bird	8	Basal diet	Liver, red blood cells	0.115	192–269
			Sodium selenite		2	308
			Selenium-yeast		2	328–439
[41]	Rodent	8	Basal diet	Heart, kidney, liver, plasma	0.02	23–179
			Sodium selenate		0.5	76–672
[42]	Rodent	9	Basal diet	Liver, plasma	0	0.6–8.7
			Sodium selenate		0.75; 0.15	4–831
[43]	Bird	12	Basal diet	Liver, muscle, plasma	0.05	0.7–9.8
			Sodium selenate		0.1; 0.2; 0.3	3–58
[44]	Rodent	6	Basal diet	Liver	0	22
			Sodium selenite		0.2; 2	743–1101
[45]	Rodent	5	Basal diet	Liver	0	21
			Sodium selenite		0.1; 2	1097
			Selenomethionine		0.1	1046
[46]	Bird	12	Basal diet	Liver	0.26	1416–2198
			Sodium selenite		0.46	3331–4144
			Selenium-yeast		0.46	3114–4771
[47]	Ruminant	2	Basal diet	Liver	0.11	40.00
			Sodium selenite		0.31	52.00
[48]	Horse	24	Basal diet	Muscle, plasma, red blood cells	0.15	7–233
			Sodium selenite		0.6	7–238
			Selenomethionine		0.6	7–360
[49]	Rodent	10	Basal diet	Brain, liver, muscle	0.004	36–105
			Sodium selenite		1.004	63–751
[50]	Bird	15	Basal diet	Gastrointestinal tract, kidney, liver	0.1	6–12
			Sodium selenite		0.5	13–24
			Selenium-yeast		0.5; 1	12–24
[51]	Rodent	2	Basal diet	Brain	0.2	32
			Selenium		0.01125	23
[52]	Rodent	40	Basal diet	Liver, muscle	0.02	2.5
			Selenium-enriched food		0.05; 0.1; 0.15	4–937
			Sodium selenite		0.05; 0.1; 0.15	4–877
			Selenomethionine		0.05; 0.1; 0.15	6–790
[53]	Ruminant	4	Basal diet	Plasma	0.15	810
			Sodium selenite		0.4	860
			Selenium-yeast		0.4	630
[54]	Rodent	5	Basal diet	Liver	0.011	40
			Sodium selenite		0.1; 0.5; 1; 2	880–1250
[55]	Rodent	6	Basal diet	Liver, lymph nodes, skin	0.005	110–675
			Sodium selenite		0.1	645–1732
[56]	Rodent	15	Basal diet	Brain, muscle, plasma, reproductive tract, spleen	0	3–250
			Sodium selenite		0.1; 4	48–686
[57]	Rodent	63	Basal diet	Brain, heart, kidney, liver, muscle, plasma, red blood cells, reproductive tract, spleen	0	2–135
			Sodium selenate		4	2–158
			Selenomethionine		0.5; 1; 2; 4	2–237
[58]	Rodent	4	Basal diet	Brain, liver	0.016	4–64
			Sodium selenite		0.1	100
[59]	Rodent	27	Basal diet	Heart, liver, thyroid	0.003	1–70
			Sodium selenite		0.024; 0.052; 0.104; 0.405	1–2020
[60]	Rodent	8	Basal diet	Liver	0.005	6–133
			Sodium selenite		0.1005	1158–1586
[61]	Rodent	4	Basal diet	Liver	0.005	7
			Sodium selenite		0.1005	1262
[62]	Rodent	8	Basal diet	Kidney, liver	0.005	19
			Sodium selenite		0.1005	500–1080
[63]	Rodent	2	Basal diet	Liver	0.005	5
			Sodium selenite		0.1005	1080
[64]	Rodent	4	Basal diet	Liver, plasma	0	13
			Sodium selenate		0.25	340–1500
[65]	Rodent	16	Basal diet	Kidney, liver	0.45	455–801
			Selenium-yeast		0.007	17–378
[66]	Rodent	6	Basal diet	Brain, kidney, liver	0	9.3
			Sodium selenite		0.25	20–310
[67]	Rodent	8	Basal diet	Liver, thyroid	0	10–80
			Sodium selenite		0.1	50-890
[68]	Rodent	68	Basal diet	Adipose tissue, gastrointestinal tract, liver, plasma, red blood cells	0	3–180
			Selenomethionine		2	10–638
[69]	Rodent	12	Basal diet	Brain, heart, kidney, liver, lung	0.015	20
			Sodium selenate		0.25	25–320
[70]	Rodent	6	Basal diet	Reproductive tract, spleen	0.01	152
			Sodium selenite		0.1; 1	357
[25]	Rodent	71	Basal diet	Adipose, adrenal, brain, diaphragm, eye, heart, gastrointestinal tract, kidney, liver, lung, muscle, oesophagus, pancreas, reproductive tract, skin, spinal cord, spleen, thymus, tongue	0	4–455
			Sodium selenate		0.1; 4	32–1502
[35]	Bird	12	Basal diet	Kidney, liver, muscle, plasma, red blood cells	0	4–143
			Selenomethionine		0.3	7–192
[71]	Rodent	36	Basal diet	Brain, gastrointestinal tract, heart, kidney, liver, lung, reproductive tract	0.012	9
			Sodium selenite		0.5	10–156
[72]	Rodent	4	Basal diet	Heart	0	5
			Sodium selenite		0.5	109

**Table 3:** Table of effects for the overall relationship between selenium supplementation and gluthathione peroxidase (GPx) activity for animal species. Dose response relationships were determined using the equation: Log GPx activity = [Constant + Form + Tissue + Animal] + [Log Dose.Form + Log Dose.Tissue + Log Dose.Animal]. Model coefficients or effects and the average standard error of difference (SED) between pairs of effects (with the minimum and maximum SEDs where appropriate) are reported.

Parameter	Constant	Slope	*n* Treatment Groups
	1.822 ^1^	0.645 ^2^	
SE	0.146	0.116	
*p*-value		<0.001	
	**Form**	**Log Dose.Form**	
Basal diet	0	0	232
Sodium selenite	0.621	−0.366	140
Selenomethionine	0.390	−0.654	99
Sodium selenate	0.241	−0.578	69
Selenium-yeast	−0.093	0.172	33
Selenium-enriched foods	1.344	0.914	20
SED (min SED; max SED)	0.17 (0.124; 0.216)	0.264 (0.111; 0.493)	
*p*-value	<0.001	<0.001	
	**Tissue**	**Log Dose.Tissue**	
Liver	0.000	0	160
Gastrointestinal tract ^3^	−0.141	0.233	68
Plasma	−0.708	−0.158	60
Muscle	−0.621	−0.302	55
Brain	0.143	−0.219	34
Heart	0.099	−0.168	41
Kidney	0.063	−0.311	48
Red blood cells ^4^	0.516	−0.324	50
Reproductive tract ^5^	0.258	−0.153	29
Spleen	0.690	−0.225	16
Thyroid	0.532	0.026	11
Adipose tissue ^6^	−0.095	0.005	7
Lung	−0.125	−0.209	9
Skin	0.121	0.008	5
SED (min SED; max SED)	0.373 (0.130; 0.756)	0.335 (0.103; 0.653)	
*p*-value	<0.001	<0.001	
	**Animal**	**Log Dose.Animal**	
Rodent ^7^	0	0	496
Bird ^8^	−0.042	−0.294	59
Horse	−0.160	−0.262	24
Ruminant ^9^	0.442	−0.502	14
SED (min SED; max SED)	0.548 (0.304; 0.729)	0.359 (0.095; 0.506)	
*p*-value	0.948	0.031	

^1^ This constant value constitutes the combined additive effect (intercept) of the combination of the first categories of Form, Tissue and Animal, namely Basal Diet, Adipose Tissue, and Bird. That is why the coefficients for each of these are zero; ^2^ This slope value constitutes the coefficient of LogDose (*i.e.*, slope) for the same combination of the first categories of Form, Tissue and Animal, namely Basal Diet, Adipose Tissue, and Bird. Includes the stomach, duodenum, jejunum, ileum, caecum, colon; ^3^ Includes blood, platelets, erythrocytes, thrombocytes; ^4^ Includes epididymis, prostate, seminal vesicles and testis. ^5^ Includes brown and white adipose tissue; ^6^ Includes mouse, rat and guinea pig; ^7^ Includes chickens and turkeys; ^8^ Includes sheep, goat and cow.

### 3.2. The Effects of Selenium Supplementation on GPx Activity in Humans

The meta-analysis identified 42 treatment groups investigating the effects of selenium supplementation on GPx activity in humans (Table 4). Selenium supplementation was either by selenium-yeast (20 treatment groups) or selenium-enriched food sources (e.g., selenium-enriched onions; 7 treatment groups). Red blood cells (38 treatment groups) and plasma (4 treatment groups) were the tissues studied.

REML analysis of this dataset showed that the overall relationship between selenium supplementation and GPx activity (ignoring other factors) could be described by the equation: Log GPx activity = 2.036 (SE 0.497) – 0.113 (SE 0.128) × Log Dose. The meta-analysis indicated that the slope of the relationship was not significantly affected by form of selenium (*p* = 0.70) or tissue studied (*p* = 0.70) (Table 5).

**Table 4:** Description of the country, form of selenium studied for selenoprotein activity with changing concentrations of selenium in the diet for investigated in human subjects.

Reference	Country	Form	Tissue	Se Dose Rate (mg/kg)	GPx Activity (nmol NADPH ox/min/mg prot)	Used in Meta-Analysis?
[73]	Australia	Basal diet	Plasma	150	70.9–76.3	Yes
		Selenium-milk				
		Selenium-yeast				
[74]	Britain	Basal diet	Plasma	-	-	No—units
[75]	UK	Basal diet	Platelet	50; 100; 200	250–340	Yes
		Selenium-onions				
		Selenium-yeast				
[76]	New Zealand	Basal diet	Blood	300	6.49	Yes
		Selenium-yeast				
[77]	New Zealand	Basal diet	Plasma, platelet, whole blood	-	-	No-data reported graphically
		Sodium selenate		-	-	
		Selenomethionine		-	-	
[78]	USA	Basal diet	Erythrocyte, plasma	-	-	No—data reported graphically
		Selenium-glycinate		-	-	
[79]	Sweden	Basal diet	Plasma	-	-	No—no control (selenium-free) group
[80]	Denmark	Basal diet	Erythocytes, plasma, thrombocytes	-	-	No—units
		Sodium selenate		-	-	
		Selenium-milk		-	-	
		Selenium-yeast		-	-	
[81]	China	Basal diet	Plasma, red blood cells	-	-	No—data reported graphically
		Sodium selenite		-	-	
		Selenium-yeast		-	-	
[82]	Italy	Basal diet	Plasma	-	-	No—units
		Sodium selenite		-	-	
[12]	UK	Basal diet	Erythocytes, platelet	50; 100; 200	43.0–12800	Yes
		Enriched Protein				
		Onions				
		Selenium-onions				
		Selenium-yeast				
[83]	Denmark	Basal diet	Erythocytes, plasma, thrombocytes	-	-	No—units
		Selenium-yeast		-	-	
[84]	USA	Basal diet	Plasma	-	-	No—data reported graphically
		Selenomethionine		-	-	
[85]	USA	Basal diet	Plasma	-	-	No—no control (selenium-free) group
[86]	China	Basal diet	Plasma	-	-	No—data reported graphically
		Sodium selenite		-	-	
		Selenomethionine		-	-	
[87]	USA	Basal diet	Plasma	-	-	No—data reported graphically
		Sodium selenite		-	-	
		Selenomethionine		-	-	
		Selenium-yeast		-	-	
[88]	Finland	Basal diet	Plasma, platelet, red blood cells	- - -	-	No—units
		Sodium selenite			-	
		Selenium-yeast			-	

**Table 5:** Table of effects for humans showing the relationship between selenium intake and Glutathione Peroxidase (GPx) activity. Dose response relationships were determined using the equation: Log GPx activity = (Constant + Form + Tissue) + (Log Dose.Form + Log Dose.Tissue). Model coefficients or effects, and the average standard error of difference (SED) between pairs of effects (with the minimum and maximum SEDs where appropriate) are reported.

Parameter	Constant	Slope	*n* Treatment Groups
	2.036 ^2^	−0.113 ^3^	
SE	0.497	0.128	
*p*-value		0.004	
	**Form**	**Log Dose.Form**	
Basal diet	0	0	15
Selenium-enriched foods	0.240	0.000	7
Selenium-yeast	0.231	0.050	20
SED (min SED; max SED)	0.154 (0.080; 0.226)	0.131	
*p*-value	0.011	0.703	
	**Tissue**	**Log Dose.Tissue**	
Red blood cells ^1^	0.0	0	38
Plasma	−0.312	−6.74 × 10^−4^	4
SED	0.984	0.019	
*p*-value	0.740	0.704	

^1^ Includes blood, platelet, erythrocyte; ^2^ This constant value constitutes the combined additive effect (intercept) of the combination of the first categories of Form and Tissue, namely Basal Diet and Red blood cells. That is why the coefficients for each of these are zero; ^3^ This slope value constitutes the coefficient of Log Dose (*i.e.*, slope) for the same combination of the first categories of Form and Tissue, namely Basal Diet and Red blood cells.

## 4. Discussion

GPx synthesis utilises selenium (via selenocysteine) and glutathione derived from transsulfuration of homocysteine; homocysteine is derived from *S*-adenosylhomocysteine, which is formed as a result of methylation reactions including methylation of selenium [31,89]. Selenium is thought to exert epigenentic effects by altering the relative concentrations of *S*-adenosyl methionine and *S*-adenosyl homocysteine, thereby modulating DNA methylation and gene transcription via the methionine-homocysteine cycle [31]. These observations demonstrate an interaction between selenium, GPx enzyme activity, and methylation, although its precise nature remains unclear.

Given the conflicting results in the literature surrounding the effects of selenium supply and selenoprotein activity, it was of interest to attempt to determine quantitatively any differences between the form of selenium and selenoprotein activity. To address one component of this interaction, this meta-analysis quantified the relationship between selenium supplementation and global GPx activity. For all tissues and animal species studied (including humans), selenium supplementation increased GPx activity. The exact relationship between selenium supply and GPx activity depended on the form of selenium used and the tissue being investigated. Importantly, the study suggests that compared with selenomethionine and selenium-yeast in particular, selenium-enriched food are more effective in increasing GPx activity in a range of tissues. These results are discussed in relationship to the role of selenium in epigenetic regulation of DNA methylation, gene expression and histone deacetylase (HDAC) inhibition.

### 4.1. Form Differences

The dose-response relationships generated in the current study suggest that selenium-enriched foods are the most effective at increasing GPx activity in a range of animal species. However, the selenium-enriched foods group had a smaller number of observations and no higher dose rates were included. Therefore, more data investigating enriched protein sources and using higher levels of selenium would be of value. Interestingly, of the three most common forms of selenium studied (*i.e.*, sodium selenate, sodium selenite and selenomethionine), the inorganic forms of selenium were more effective at increasing GPx activity compared with selenomethionine (see Table 3, Log Dose.Form interactions). As the form of selenium fed was found to affect the relationship between selenium supply and GPx activity, this may explain the apparent contradictory information in the literature concerning the effects of selenium supply on GPx activity [21,90,91,92,93].

Much discussion in the literature exists in terms of the bioavailability of different forms of selenium and their ability to raise selenoprotein activity in humans [75,87]. Traditionally, inorganic forms of selenium (sodium selenite and sodium selenate) have been thought to be less effective at increasing biomarkers of selenium status than organic forms (selenomethionine), based on selenium concentrations in plasma [86,87]. However, a previous meta-analysis suggested that inorganic forms increased platelet GPx activity more effectively than organic forms [94]. More recently, it has been suggested that the response to supplementation varies with baseline status and that this is form dependent. For example, selenomethionine increases plasma selenium in people with either low or optimal selenium status but selenite has little effect unless the subjects are of low status [84]. Recent results have shown that selenium glycinate (an inorganic form of selenium) increased GPx activity in men [78]. Additionally, red blood cell GPx activity increased with short-term (4 weeks) selenate supplementation whereas organic selenium (selenium enriched milk and selenium-yeast) produced no effect in humans [80]. Selenium-yeast increased GPx1 and GPx4 activity in platelets after 12 weeks of supplementation in humans [12]. Interestingly, selenium-enriched onions decreased GPx4 activity after 10 weeks of supplementation [12]. In humans with optimal baseline selenium status, selenomethionine supplementation (50–200 μg/day for 12 months) did not affect GPx3 activity or selenoprotein P levels [84]. Some of these differences may reflect non-specific incorporation of selenomethionine into proteins such as albumin. Additionally, data shows that the chemical form of selenium at nutritional doses can affect the absorption and retention of selenium in a human intestinal Caco-2 cell model [95]. These authors showed that selenomethionine, and digested selenium enriched yeast were transported at comparable efficacy from the apical to basolateral sides, each being about three-fold that of selenite; but that these effects are not directly correlated to the potential to support GPx activity [95].

There is increasing interest in selenium-enriched foods and their impacts on human health, due to the link between selenium status and cancer risk. In the current analysis, selenium-enriched protein sources (e.g., milk, meat; [23,52,73]) enhanced the rate of increase in GPx activity compared to selenium-yeast; unfortunately we were unable to include other inorganic forms of selenium into the meta-analysis due to the format in which GPx activity was reported (graphs, or units). While selenium-enriched rice increased serum GPx activity in humans with normal selenium [96], selenium-enriched onions did not affect platelet GPx activity [75]. Selenium enriched probiotics (containing >90% organic Se; of the organic Se, >75% is selenomethionine) increased GPx activity in the eggs of laying hens [97] and in the blood [98,99] and tissues [99] of pigs. It is important to note that selenium-enriched foods contain multiple forms of selenium, including organic (selenomethionine) and inorganic forms (sodium selenite and sodium selenate) [75]. The effectiveness of selenium-enriched foods in increasing GPx activity observed in the present study is consistent with earlier studies in which selenium enriched milk was more effective than selenium-yeast and more protective against colon tumouriogenesis in mouse models [100]. Furthermore, when mice were fed selenium in the forms of sodium selenite, selenomethionine and selenium-yeast only the selenium-yeast tended to reduce DNA oxidation [101]. Thus, considering both previous and present work, although there are complex and contradictory findings as regards the relative effectiveness of different forms of dietary selenium on GPx activities, in general the greatest effects in both animals and humans are found with protein-based sources of selenium.

#### 4.1.1. Form Effects on Epigenetic Regulation

Epigenetic modifications of genes in response to selenium supplementation have been investigated *in vivo* and *in vitro*, with the majority of studies using cell lines to investigate the epigenetic regulation of genes in response to selenium. Of the selenoproteins, only the epigenetic regulation of GPx1 [102,103] and GPx3 [104] have been investigated, but not in relation to selenium supplementation. In terms of the effects of selenium supply on epigenetic regulation, both inorganic and organic forms of selenium have been used to investigate global DNA methylation and gene specific methylation. Investigations on the effect of selenium supplementation have been reported to a lesser extent for HDAC activity and miRNA.

Sodium selenite reduced global DNA methylation in the heart of mice [89]. In the liver and colon mucus of the rat, dietary selenomethionine supplementation has been shown to cause global DNA hypomethylation [22]. Sodium selenite also decreased DNA methylation in human prostate cancer cells [105]. Selenomethione increased DNA methylation of the p53 gene but did not affect the methylation of β-actin gene in the rat [22]. Low levels of methylselenocysteine resulted in increased methylation of the von Hippel-Lindau (VHL) gene *in vitro* [106]. Selenium methylselenocysteine and selenite supplementation did not affect the methylation of ESR1, p16 (INK4A) or of LINE-1 *in vitro* [107]. Seleno-DL-methionine did not affect the methylation status of glutathione sulfotransferase pi (GSTP1) nor Ras associated family 1A (RASSF1A) genes *in vitro* [108]. DNA methylation profile between Keshan Disease patients and normal individuals showed that selenium deficiency decreased methylation of CpG islands in promoter regions of TLR2 and ICAM1, in agreement with results from *in vivo* and *in vitro* models of the disease [109].

Methylselenocysteine and selenomethionine are organoselenium compounds that can be converted to beta-methylselenopyruvate and alpha-keto-gamma-methylselenobutyrate. These alpha-keto acid metabolites share structural features with butyrate (an HDAC inhibitor) [110]. Beta-methylselenopyruvate and alpha-keto-gamma-methylselenobutyrate alter HDAC activity and histone acetylation status *in vitro* [110]. Supplementation of methylseleninic acid inhibited HDAC activity in B-cell lymphoma cell lines [111] and in esophageal squamous cell carcinoma cell lines [112]. Se-Methyl-l-selenocysteine and selenomethionine had no effect on HDAC activity while beta-methylselenopyruvate and alpha-keto-gamma-methylselenobutyrate inhibited HDAC activity *in vitro* [113]. Selenium-enriched milk has been shown to inhibit HDAC activity *in vivo* [114]. Furthermore, sodium selenite has recently been shown to regulate the expression of GPx2 and SEPSH2 via miRNA [20]. These observations collectively indicate a potentially important, albeit somewhat unclear, role of several epigenetic mechanisms in modulating the effects of selenium.

### 4.2. Selenium Supply and GPx Activity in Humans

To our knowledge, there have been two attempts to determine the relationship between selenium supplementation and GPx activity in humans using meta-analysis [94,115], with an additional study published in Chinese that showed supplementation with organic selenium increases the activity of GPx in healthy adults (abstract only [116]). However, no studies have attempted to quantify the relationship between GPx activity and selenium supplementation. Additionally, Ashton *et al.* [115] excluded selenite on the basis of a supplementation study in the USA which appeared to supplement human subjects who were already selenium replete [87]. This is despite literature supporting an effect of selenite supplementation (100 μg/day) on selenium status in humans [117]. In humans, supplementation of selenomethionine (100 μg) increased whole blood GPx activity [118]. It has been suggested that GPx activity in humans is not sensitive to changes in selenium supply unless the people had low baseline levels of selenium [94]. However, more recently, studies have shown that selenium-enriched milk protein (dairy-selenium) or selenium-rich yeast (yeast-selenium) plasma GPx activity was not changed in humans with low plasma selenium status [73] and in men with high and low selenium status neither prostate tissue nor serum selenium concentrations were associated with prostate tissue GPx activity [119]. Additionally, in healthy males with higher selenium status supplementation with selenized yeast (Selplex, 200 μg/day) reduced red blood cell GPx activity [120].

The current meta-analysis suggests that in humans GPx activity increases with selenium supply (with red blood cells being more responsive than plasma), in agreement with previous observations [115]. This is not unexpected since GPx1, which is more sensitive than GPx3 to selenium supply, is the predominant form in red blood cells and GPx3 the form in plasma. Predicted dose-response relationships suggest the same would hold true of other tissues; however these relationships need to be strengthened by more information on the effects of selenium supplementation and the various GPx activities in other tissues [115].

### 4.3. GPx Activity/Expression as a Biomarker of Selenium Requirements

Currently, there is debate as to the appropriateness of GPx1 and GPx3 activities or mRNA levels as biomarkers for selenium requirements [10,11,12,13]. Interestingly, results from single studies indicate that GPx activity is not an appropriate biomarker for selenium status [10,11,12,13] whereas the conclusion from a systematic review was that GPx activity may have a role as a biomarker of selenium status [115].

Initially, we had planned to assess the effects of selenium supplementation on mRNA expression of GPx, however our literature search identified that few publications provided quantitative effects of selenium supplementation on GPx mRNA. Therefore, we were unable to include this parameter in our study and instead focussed on GPx activity. Although the activity of selenoproteins does not always reflect changes in mRNA levels [83], previous work indicates that levels of selenoprotein mRNAs respond to selenium supplementation in a differential manner [11,23] that partly reflects the selenoprotein hierarchy due to different effects of low Se supply on mRNA [73]. Interestingly, the complexity of selenium form also carries through to mRNA expression. For example, selenium-enriched onions (12 weeks study with humans) increased the expression of selenoprotein W1, S1 and R mRNA in humans [12] and selenium-enriched milk increased the expression of GPx1 and GPx2 mRNA in humans (compared to selenium-yeast) after 6 weeks of supplementation [73]. However, there was no effect of short-term (4 weeks) inorganic (selenite) and organic (selenium-yeast and selenium-enriched milk) selenium supplementation on the expression of selenoprotein mRNA (GPx1, thioredoxin reductase-1 and selenoprotein P) in leucocytes in humans [80]. More research in this area is needed to clarify this relationship.

### 4.4. GPx Activity Is Dependent on the Tissue Studied

The current analysis identifies that there is a hierarchy in terms of the responsiveness of tissue GPx activity to selenium supplementation [31], in agreement with previous studies reporting the effects of GPx activity in multiple tissue beds concurrently [25,57,71] and consistent with the previously described selenoprotein hierarchy [68,121,122,123]. For example, Sun *et al.* [25] observed that GPx activity was present in 28 tissues in the rat (compared to selenoprotein W which was not detected in all tissues) and that different tissues react differently to alterations in selenium supply. Other studies have shown that supplementation with selenomethionine had no effects on the activity of GPx in brain, testis, heart, muscle and plasma whereas spleen and red blood cells had increased GPx activity [57].

In another study [68], it was observed that supplementation with selenomethionine increased the activity of GPx in the gastrointestinal tract, liver and adipose tissue. The present study also indicates that GPx activity in the gastrointestinal tract is sensitive to selenium supply and this may reflect the presence of GPx2, a selenoprotein that ranks highly in the selenoprotein hierarchy. In addition, the current dataset indicates that there are no decreases in tissue GPx activity associated with higher levels of selenium supplementation. However, this could be due to the relatively small number of treatments investigating the effects of high levels of selenium supplementation.

### 4.5. Limitations of the Meta-Analysis

As there was limited information on all forms of GPx (*i.e.*, GPx 1–8), the present analysis considered GPx1–8 isoforms occurring in a tissue as a single, combined variable of global GPx activity. Given that there are differences in terms of biochemistry, physiology and metabolism of the different GPx, this affects how the data can be interpreted, and more information on individual forms would be of value. Additionally, the present study did not consider selenoproteins other than GPx1–8, including selenoprotein P which is known to respond to selenium supplementation in a dose-responsive manner [75]. This largely reflects both the relatively few publications in this area with data on these other selenoproteins in the quantitative format required for meta-analysis and the bulk of literature with appropriate data focussing on GPx activity. Human studies suggest that both GPx3 and selenoprotein P levels in plasma exhibit a linear relation to selenium intake following supplementation but that GPx3 reaches a maximal level at a lower selenium intake than selenoprotein P [87,124].

While we identified 17 publications that reported GPx activity in response to selenium supplementation in humans (see Table S2), for a variety of reasons, including issues around units (not reported or compatible), only four publications (42 treatment groups) were included in the current meta-analysis. With such small numbers of publications over a small range of selenium forms (selenium-enriched protein and selenium-yeast), this analysis can therefore only be regarded as preliminary in terms of the situation in humans. A recent review assessing the selenium status of humans indicated a number of key issues in terms of interpreting selenium supplementation data which are in agreement with the findings from the current analysis, namely a lack of consistency between the units of measurement for key analyses including selenium concentration in urine and selenoprotein activity [115]. Inconsistency in units measuring selenoprotein activity has been pointed out previously [125], and it appears that the unit of activity may impact the interpretation of the data. For example, when GPx activity in platelets was measured in μmol NADPH oxidised/min/g protein it showed a significant response to selenium supplementation whereas those measured in nmol NADPH oxidised/min/mL plasma did not [115].

In many publications, the basal diet was not analysed for the final selenium concentration. Therefore, for the purposes of this analysis we assumed that all basal diets (control diets) contained no selenium. While this assumption may not be completely accurate (for example, the basal diet of Sun *et al.* [25] contained 4 ng selenium per kg diet), it enables us to determine the dose-response relationships between increases in selenium intake and GPx activity. Additionally it is highly probable that the content of the basal diet is much less than the amount of selenium added (e.g., 0.1 or 4 mg/kg) [25].

The present study excluded data sets from “depletion-repletion” studies and it assumed that at the start of the study all animals had an adequate selenium status. Results from individual human studies suggest that baseline selenium status may affect the relationship between GPx activity and selenium supply. Selenomethionine increases plasma selenium in people with both low and optimal selenium status but selenite has little effect unless subjects are of low status [84]. For example, selenomethionine did not affect GPx3 activity in humans with optimal selenium intake [84], whereas selenite increases plasma GPx activity and plasma selenium concentration in humans with sub-optimal baseline selenium status [117]. In addition, our meta-analysis shows that plasma GPx activity is relatively resistant to selenium supplementation and that activity in other tissues is more sensitive and may be more appropriate to measure as a biomarker of status.

As the overall meta-analysis includes data on GPx activity in non-blood tissues, it is also possible that the selenium-enriched proteins are effective in supplying selenium to the non-blood tissues for humans. Indeed, supplementation with selenium-enriched dairy protein increased rectal biopsy GPx activity without concurrent increases in plasma GPx activity [50]. Overall, the relative effectiveness of the different forms at delivering selenium to non-blood tissues in humans with suboptimal selenium status is not clear and should be investigated further.

## 5. Conclusions

In conclusion, this study indicates that in animals selenium-enriched food is more effective in increasing GPx activity in a range of tissues compared with other forms of selenium including selenomethionine. Such an analysis will provide essential information regarding selenium dosage and form a basis for subsequent studies that aim to investigate how selenium interacts with epigenetic mechanisms to exert its effects, in particular those which involve GPx enzymes and other selenoproteins.

## Supplementary Files

Supplementary File 1
