# Supplementary material for: Selenium-Enriched Foods Are More Effective at Increasing Glutathione Peroxidase (GPx) Activity Compared with Selenomethionine: A Meta-Analysis
Source: Nutrients. 2014 Sep 29;6(10):4002–31. doi: 10.3390/nu6104002 (PMC4210904; doi:10.3390/nu6104002)
Supplement: Supplementary File 1 [file nutrients-06-04002-s001.docx]

**Supplementary Information**

**Table S1.** Description of the form of selenium studied for selenoprotein activity with changing concentrations of selenium in the diet for animal species. The table indicates the number of treatment groups for each publication.

| **References** | **Animal Species** | ***n* Treatment Groups** | **Diet Form** | **Tissue** |
| --- | --- | --- | --- | --- |
| [1] | Rodent | 5 | Basal diet | Gastrointestinal tract |
|  |  |  | Selenium-enriched food |  |
|  |  |  | Selenium-yeast |  |
| [2] | Rodent | 45 | Basal diet | Heart, kidney, liver, plasma, red blood cells |
|  |  |  | Sodium selenite |  |
|  |  |  | Selenomethionine |  |
|  |  |  | Selenium-yeast |  |
| [3] | Rodent | 16 | Basal diet | Plasma, red blood cells |
|  |  |  | Sodium selenite |  |
| [4] | Ruminant | 8 | Basal diet | Muscle, red blood cells |
|  |  |  | Sodium selenite |  |
|  |  |  | Selenium-yeast |  |
| [5] | Rodent | 4 | Basal diet | Liver |
|  |  |  | Sodium selenite |  |
| [6] | Bird | 8 | Basal diet | Liver, red blood cells |
|  |  |  | Sodium selenite |  |
|  |  |  | Selenium-yeast |  |
| [7] | Rodent | 8 | Basal diet | Heart, kidney, liver, plasma |
|  |  |  | Sodium selenate |  |
| [8] | Rodent | 9 | Basal diet | Liver, plasma |
|  |  |  | Sodium selenate |  |
| [9] | Bird | 12 | Basal diet | Liver, muscle, plasma |
|  |  |  | Sodium selenate |  |

**Table S1.** *Cont.*

| **References** | **Animal Species** | ***n* Treatment Groups** | **Diet Form** | **Tissue** |
| --- | --- | --- | --- | --- |
| [10] | Rodent | 6 | Basal diet | Liver |
|  |  |  | Sodium selenite |  |
| [11] | Rodent | 5 | Basal diet | Liver |
|  |  |  | Sodium selenite |  |
|  |  |  | Selenomethionine |  |
| [12] | Bird | 12 | Basal diet | Liver |
|  |  |  | Sodium selenite |  |
|  |  |  | Selenium-yeast |  |
| [13] | Ruminant | 2 | Basal diet | Liver |
|  |  |  | Sodium selenite |  |
| [14] | Horse | 24 | Basal diet | Muscle, plasma, red blood cells |
|  |  |  | Sodium selenite |  |
|  |  |  | Selenomethionine |  |
| [15] | Rodent | 10 | Basal diet | Brain, liver, muscle |
|  |  |  | Sodium selenite |  |
| [16] | Bird | 15 | Basal diet | Gastrointestinal tract, kidney, liver |
|  |  |  | Sodium selenite |  |
|  |  |  | Selenium-yeast |  |
| [17] | Rodent | 2 | Basal diet | Brain |
|  |  |  | Selenium |  |
| [18] | Rodent | 40 | Basal diet | Liver, muscle |
|  |  |  | Selenium-enriched food |  |
|  |  |  | Sodium selenite |  |
|  |  |  | Selenomethionine |  |
| [19] | Ruminant | 4 | Basal diet | Plasma |
|  |  |  | Sodium selenite |  |
|  |  |  | Selenium-yeast |  |

**Table S1.** *Cont.*

| **References** | **Animal Species** | ***n* Treatment Groups** | **Diet Form** | **Tissue** |
| --- | --- | --- | --- | --- |
| [20] | Rodent | 5 | Basal diet | Liver |
|  |  |  | Sodium selenite |  |
| [21] | Rodent | 6 | Basal diet | Liver, lymph nodes, skin |
|  |  |  | Sodium selenite |  |
| [22] | Rodent | 15 | Basal diet | Brain, muscle, plasma, reproductive tract, spleen |
|  |  |  | Sodium selenite |  |
| [23] | Rodent | 63 | Basal diet | Brain, heart, kidney, liver, muscle, plasma, red blood cells,  reproductive tract, spleen |
|  |  |  | Sodium selenate |  |
|  |  |  | Selenomethionine |  |
| [24] | Rodent | 4 | Basal diet | Brain, liver |
|  |  |  | Sodium selenite |  |
| [25] | Rodent | 27 | Basal diet | Heart, liver, thyroid |
|  |  |  | Sodium selenite |  |
| [26] | Rodent | 8 | Basal diet | Liver |
|  |  |  | Sodium selenite |  |
| [27] | Rodent | 4 | Basal diet | Liver |
|  |  |  | Sodium selenite |  |
| [28] | Rodent | 8 | Basal diet | Kidney, liver |
|  |  |  | Sodium selenite |  |
| [29] | Rodent | 2 | Basal diet | Liver |
|  |  |  | Sodium selenite |  |
| [30] | Rodent | 4 | Basal diet | Liver, plasma |
|  |  |  | Sodium selenate |  |
| [31] | Rodent | 16 | Basal diet | Kidney, liver |
|  |  |  | Selenium-yeast |  |
| [32] | Rodent | 6 | Basal diet | Brain, kidney, liver |
|  |  |  | Sodium selenite |  |

**Table S1.** *Cont.*

| **References** | **Animal Species** | ***n* Treatment Groups** | **Diet Form** | **Tissue** |
| --- | --- | --- | --- | --- |
| [33] | Rodent | 8 | Basal diet | Liver, thyroid |
|  |  |  | Sodium selenite |  |
| [34] | Rodent | 68 | Basal diet | Adipose tissue, gastrointestinal tract, liver, plasma, red blood cells |
|  |  |  | Selenomethionine |  |
| [35] | Rodent | 12 | Basal diet | Brain, heart, kidney, liver, lung |
|  |  |  | Sodium selenate |  |
| [36] | Rodent | 6 | Basal diet | Reproductive tract, spleen |
|  |  |  | Sodium selenite |  |
| [37] | Rodent | 71 | Basal diet  Sodium selenate | Adipose, adrenal, brain, diaphragm, eye, heart, gastrointestinal tract, kidney, liver, lung, muscle, oesophagus, pancreas, reproductive tract, skin, spinal cord, spleen, thymus, tongue |
| [38] | Bird | 12 | Basal diet | Kidney, liver, muscle, plasma, red blood cells |
|  |  |  | Selenomethionine |  |
| [39] | Rodent | 36 | Basal diet | Brain, gastrointestinal tract, heart, kidney, liver, lung, reproductive tract |
|  |  |  | Sodium selenite |  |
| [40] | Rodent | 4 | Basal diet | Heart |
|  |  |  | Sodium selenite |  |

**Table S2.** Description of the country, form of selenium studied for selenoprotein activity with changing concentrations of selenium in the diet for investigated in human subjects.

| **References** | **Country** | **Form** | **Tissue** | **Used in Meta-Analysis?** |
| --- | --- | --- | --- | --- |
| [41] | Australia | Basal diet | Plasma | Yes |
|  |  | Selenium-milk |  |  |
|  |  | Selenium-yeast |  |  |
| [42] | Britian | Basal diet1 | Plasma | No—units |
| [43] | UK | Basal diet | Platelet | Yes |
|  |  | Selenium-onions |  |  |
|  |  | Selenium-yeast |  |  |
| [44] | New Zealand | Basal diet | Blood | Yes |
|  |  | Selenium-yeast |  |  |
| [45] | New Zealand | Basal diet | Plasma, platelet, whole blood | No—data reported graphically |
|  |  | Sodium selenate |  |  |
|  |  | Selenomethionine |  |  |
| [46] | USA | Basal diet | Erythrocyte, plasma | No—data reported graphically |
|  |  | Selenium-glycinate |  |  |
| [47] | Sweden | Basal diet1 | Plasma | No—no control (selenium-free) group |
| [48] | Denmark | Basal diet | Erythocytes, plasma, thrombocytes | No—units |
|  |  | Sodium selenate |  |  |
|  |  | Selenium-milk |  |  |
|  |  | Selenium-yeast |  |  |
| [49] | China | Basal diet | Plasma, red blood cells | No—data reported graphically |
|  |  | Sodium selenite |  |  |
|  |  | Selenium-yeast |  |  |
| [50] | Italy | Basal diet | Plasma | No—units |
|  |  | Sodium selenite |  |  |

**Table S2.** *Cont.*

| **References** | **Country** | **Form** | **Tissue** | **Used in Meta-Analysis?** |
| --- | --- | --- | --- | --- |
| [51] | UK | Basal diet | Erythrocytes, platelet | Yes |
|  |  | Enriched Protein |  |  |
|  |  | Onions |  |  |
|  |  | Selenium-onions |  |  |
|  |  | Selenium-yeast |  |  |
| [52] | Denmark | Basal diet | Erythrocytes, plasma, thrombocytes | No—units |
|  |  | Selenium-yeast |  |  |
| [53] | USA | Basal diet | Plasma | No—data reported graphically |
|  |  | Selenomethionine |  |  |
| [54] | USA | Basal diet1 | Plasma | No—no control (selenium-free) group |
| [55] | China | Basal diet | Plasma | No—data reported graphically |
|  |  | Sodium selenite |  |  |
|  |  | Selenomethionine |  |  |
| [56] | USA | Basal diet | Plasma | No—data reported graphically |
|  |  | Sodium selenite |  |  |
|  |  | Selenomethionine |  |  |
|  |  | Selenium-yeast |  |  |
| [57] | Finland | Basal diet | Plasma, platelet, red blood cells | No—units |
|  |  | Sodium selenite |  |  |
|  |  | Selenium-yeast |  |  |

References

1. Hu, Y.; McIntosh, G.H.; Le Leu, R.K.; Young, G.P. Selenium-enriched milk proteins and selenium yeast affect selenoprotein activity and expression differently in mouse colon. *Br. J. Nutr.* **2010**, *104*, 17–23.
2. Smith, A.M.; Picciano, M.F. Relative bioavailability of seleno-compounds in the lactating rat.
   *J. Nutr.* **1987**, *117*, 725–731.
3. Smith, A.M.; Picciano, M.F. Evidence for increased selenium requirement for the rat during pregnancy and lactation. *J. Nutr.* **1986**, *116*, 1068–1079.
4. Juniper, D.T.; Phipps, R.H.; Ramos-Morales, E.; Bertin, G. Effect of dietary supplementation with selenium-enriched yeast or sodium selenite on selenium tissue distribution and meat quality in beef cattle. *J. Anim. Sci.* **2008**, *86*, 3100–3109.
5. Tabatabaei, N.; Jamalian, J.; Owji, A.A.; Ramezani, R.; Karbalaie, N.; Rajaeifard, A.R. Effects of dietary selenium supplementation on serum and liver selenium, serum malondialdehyde
   and liver glutathione peroxidase activity in rats consuming thermally oxidized sunflower oil.
   *Food Chem. Toxicol.* **2008**, *46*, 3501–3505.
6. Upton, J.R.; Edens, F.W.; Ferket, P.R. The effects of dietary oxidized fat and selenium source on performance, glutathione peroxidase, and glutathione reductase activity in broiler chickens.
   *J. Appl. Poult. Res.* **2009**, *18*, 193–202.
7. Zhu, Z.; Kimura, M.; Itokawa, Y. Effect of selenium and protein deficiency on selenium and glutathione peroxidase in rats. *Biol. Trace Elem. Res.* **1993**, *36*, 15–23.
8. Mueller, A.S.; Klomann, S.D.; Wolf, N.M.; Schneider, S.; Schmidt, R.; Spielmann, J.; Stangl, G.; Eder, K.; Pallauf, J. Redox regulation of protein tyrosine phosphatase 1B by manipulation of dietary selenium affects the triglyceride concentration in rat liver. *J. Nutr.* **2008**, *138*, 2328–2336.
9. Mueller, A.S.; Fischer, J.; Most, E.; Pallauf, J. Investigation into selenium requirement of growing turkeys offered a diet supplemented with two levels of vitamin E. *J. Anim. Physiol. Anim. Nutr.* **2009**, *93*, 313–324.
10. Uthus, E.; Ross, S. Dietary selenium affects homoscysteine metabolism differently in Fisher-344 rats and CD-1 mice. *J. Nutr.* **2007**, *137*, 1132–1136.
11. Davis, C.; Uthus, E.; Finley, J. Dietary selenium and arsenic affect DNA methylation *in vitro* in Caco-2 cells and *in vitro* in rat liver and colon. *J. Nutr.* **2000**, *130*, 2903–2909.
12. Mahmoud, K.Z.; Edens, F.W. Influence of selenium sources on age-related and mild heat
    stress-related changes of blood and liver glutathione redox cycle in broiler chickens (*Gallus domesticus*). *Comp. Biochem. Physiol.* **2003**, *136*, 921–934.
13. Chadio, S.E.; Kotsampasi, B.M.; Menegatos, J.G.; Zervas, G.P.; Kalogiannis, D.G. Effect of selenium supplementation on thyroid hormone levels and selenoenzyme activities in growing lambs. *Biol. Trace Elem. Res.* **2006**, *109*, 145–154.
14. Richardson, S.M.; Siciliano, P.D.; Engle, T.E.; Larson, C.K.; Ward, T.L. Effect of selenium supplementation and source on the selenium status of horses. *J. Anim. Sci.* **2006**, *84*, 1742–1748.
15. Sun, Y.; Butler, J.A.; Whanger, P.D. Glutathione peroxidase activity and selenoprotein W levels in different brain regions of selenium-depleted rats. *J. Nutr. Biochem.* **2001**, *12*, 88–94.
16. Petrovič, V.; Boldižárová, K.; Faix, S.; Mellen, M.; Arpasova, H.; Leng, L’. Antioxidant and selenium status of laying hens fed with diets supplemented with selenite or Se-yeast. *J. Anim. Feed Sci.* **2006**, *15*, 435–444.
17. Castaño, A.; Ayala, A.; Rodriguez-Gomez, J.A.; De La Cruz, C.P.; Revilla, E.; Cano, J.; Machado, A. Increase in dopamine turnover and tyrosine hydroxylase enzyme in hippocampus of rats fed on low selenium diet. *J. Neurosci. Res.* **1995**, *42*, 684–691.
18. Butler, J.A.; Deagen, J.T.; Van Ryssen, J.B.J.; Rowe, K.E.; Whanger, P.D. Bioavailability to rats of selenium in ovine muscle, liver and hemoglobin. *Nutr. Res.* **1991**, *11*, 1293–1305.
19. Chung, J.Y.; Kim, J.H.; Ko, Y.H.; Jang, I.S. Effects of dietary supplemented inorganic and organic selenium on antioxidant defense systems in the intestine, serum, liver and muscle of Korean native goats. *Asian-Austr. J. Anim. Sci.* **2007**, *20*, 52–59.
20. Debski, B.; Milner, J.A. Influence of dietary selenium and cytochrome P450 modifiers on liver selenium content and cytochrome P450 activity in rats. *Trace Elem. Electrolytes* **2002**, *19*, 209–214.
21. Rafferty, T.S.; Norval, M.; El-Ghorr, A.; Beckett, G.; Arthur, J.; Nicol, F.; Hunter, J.; McKenzie, R. Dietary selenium levels determine epidermal langerhans cell numbers in mice. *Biol. Trace
    Elem. Res.* **2003**, *92*, 161–171.
22. Yeh, J.Y.; Vendeland, S.C.; Gu, Q.; Butler, J.A.; Ou, B.R.; Whanger, P.D. Dietary selenium increases selenoprotein W levels in rat tissues. *J. Nutr.* **1997**, *127*, 2165–2172.
23. Gu, Q.-P.; Xia, Y.-M.; Ha, P.-C.; Butler, J.A.; Whanger, P.D. Distribution of selenium between plasma fractions in guinea pigs and humans with various intakes of dietary selenium. *J. Trace Elem. Med. Biol.* **1998**, *12*, 8–15.
24. Chanoine, J.P.; Safran, M.; Farwell, A.P.; Tranter, P.; Ekenbarger, D.M.; Dubord, S.; Alex, S.; Arthur, J.R.; Beckett, G.J.; Braverman, L.E.; *et al.* Selenium deficiency and type II 5’-deiodinase regulation in the euthyroid and hypothyroid rat: Evidence of a direct effect of thyroxine. *Endocrinology* **1992**, *131*, 479–484.
25. Bermano, G.; Nicol, F.; Dyer, J.A.; Sunde, R.A.; Beckett, G.J.; Arthur, J.R.; Hesketh, J.E.
    Tissue-specific regulation of selenoenzyme gene expression during selenium deficiency in rats. *Biochem. J.* **1995**, *311*, 425–430.
26. Arthur, J.R.; Morrice, P.C.; Nicol, F.; Beddows, S.E.; Boyd, R.; Hayes, J.D.; Beckett, G.J.
    The effects of selenium and copper deficiencies on glutathione *S*-transferase and glutathione peroxidase in rat liver. *Biochem. J.* **1987**, *248*, 539–544.
27. Beckett, G.J.; Beddows, S.E.; Morrice, P.C.; Nicol, F.; Arthur, J.R. Inhibition of hepatic deiodination of thyroxine is caused by selenium deficiency in rats. *Biochem. J.* **1987**, *248*, 443–447.
28. Beckett, G.J.; Nicol, F.; Proudfoot, D.; Dyson, K.; Loucaides, G.; Arthur, J.R. The changes in hepatic enzyme expression caused by selenium deficiency and hypothyroidism in rats are produced by independent mechanisms. *Biochem. J.* **1990**, *266*, 743–747.
29. Beckett, G.J.; Russell, A.; Nicol, F.; Sahu, P.; Wolf, C.R.; Arthur, J.R. Effect of selenium deficiency on hepatic type I 5-iodothyronine deiodinase activity and hepatic thyroid hormone levels in the rat. *Biochem. J.* **1992**, *282*, 483–486.
30. Chittum, H.S.; Hill, K.E.; Carlson, B.A.; Lee, B.J.; Burk, R.F.; Hatfield, D.L. Replenishment of selenium deficient rats with selenium results in redistribution of the selenocysteine tRNA population in a tissue specific manner. *Biochim. Biophys. Acta* **1997**, *1359*, 25–34.
31. Nakane, T.; Asayama, K.; Kodera, K.; Hayashibe, H.; Uchida, N.; Nakazawa, S. Effect of selenium deficiency on cellular and extracellular glutathione peroxidases: Immunochemical detection and mRNA analysis in rat kidney and serum. *Free Radic. Biol. Med.* **1998**, *25*, 504–511.
32. Hill, K.E.; McCollum, G.W.; Boeglin, M.E.; Burk, R.F. Thioredoxin reductase activity is decreased by selenium deficiency. *Biochem. Biophys. Res. Commun.* **1997**, *234*, 293–295.
33. Mitchell, J.H.; Nicol, F.; Beckett, G.J.; Arthur, J.R. Selenoprotein expression and brain development in preweanling selenium- and iodine-deficient rats. *J. Mol. Endocrinol.* **1998**, *20*, 203–210.
34. Reddy, K.; Tappel, A.L. Effect of dietary selenium and autoxidized lipids on the glutathione peroxidase system of gastrointestinal tract and other tissues in the rat. *J. Nutr.* **1974**, *104*,
    1069–1078.
35. Moskovitz, J. Prolonged selenium-deficient diet in MsrA knockout mice causes enhanced oxidative modification to proteins and affects the levels of antioxidant enzymes in a tissue-specific manner. *Free Radic. Res.* **2007**, *41*, 162–171.
36. Berggren, M.M.; Mangin, J.F.; Gasdaska, J.R.; Powis, G. Effect of selenium on rat thioredoxin reductase activity: Increase by supranutritional selenium and decrease by selenium deficiency. *Biochem. Pharmacol.* **1999**, *57*, 187–193.
37. Sun, Y.; Ha, P.C.; Butler, J.A.; Ou, B.R.; Yeh, J.-Y.; Whanger, P. Effect of Dietary Selenium on Selenoprotein W and Glutathione Peroxidase in 28 Tissues of the Rat. *J. Nutr. Biochem.* **1998**, *9*, 23–27.
38. Toshiro, A.; Moriyuki, S.; Toshinori, S.; Shigekatsu, M.; Takeo, S.; Naoyuki, T.; Tsutomu, K. Glutathione peroxidase activity in tissues of chickens supplemented with dietary selenium.
    *Comp. Biochem. Physiol.* **1994**, *107*, 245–248.
39. Masukawa, T.; Nishimura, T.; Iwata, H. Differential changes of glutathione *S*-transferase activity by dietary selenium. *Biochem. Pharmacol.* **1984**, *33*, 2635–2639.
40. Xia, Y.; Hill, K.E.; Burk, R.F. Effect of selenium deficiency on Hydroperoxide-Induced glutathione release from the isolated perfused rat heart. *J. Nutr.* **1985**, *115*, 733–742.
41. Hu, Y.; McIntosh, G.H.; Le Leu, R.K.; Upton, J.M.; Woodman, R.J.; Young, G.P. The influence of selenium-enriched milk proteins and selenium yeast on plasma selenium levels and rectal selenoprotein gene expression in human subjects. *Br. J. Nutr.* **2011**, *106*, 1–11.
42. Sunde, R.A.; Paterson, E.; Evenson, J.K.; Barnes, K.M.; Lovegrove, J.A.; Gordon, M.H. Longitudinal selenium status in healthy British adults: Assessment using biochemical and molecular biomarkers. *Br. J. Nutr.* **2008**, *2008*, S37–S47.
43. Hurst, R.; Armah, C.N.; Dainty, J.R.; Hart, D.J.; Teucher, B.; Goldson, A.J.; Broadley, M.R.; Motley, A.K.; Fairweather-Tait, S.J. Establishing optimal selenium status: Results of a randomized, double-blind, placebo-controlled trial. *Am. J. Clin. Nutr.* **2010**, *91*, 923–931.
44. Karunasinghe, N.; Ferguson, L.R.; Tuckey, J.; Masters, J. Hemolysate thioredoxin reductase and glutathione peroxidase activities correlate with serum selenium in a group of New Zealand men at high prostate cancer risk. *J. Nutr.* **2006**, *136*, 2232–2235.
45. Thomson, C.D.; Robinson, M.F.; Butler, J.A.; Whanger, P.D. Long-term supplementation with selenate and selenomethionine: Selenium and glutathione peroxidase (EC 1.11.1.9) in blood components of New Zealand women. *Br. J. Nutr.* **1993**, *69*, 577–588.
46. Zhang, W.; Joseph, E.; Hitchcock, C.; DiSilvestro, R.A. Selenium glycinate supplementation increases blood glutathione peroxidase activities and decreases prostate-specific antigen readings in middle-aged US men. *Nutr. Res.* **2011**, *31*, 165–168.
47. Åkesson, B.; Huang, W.; Persson-Moschos, M.; Marchaluk, E.; Jacobsson, L.; Lindgärde, F. Glutathione peroxidase, selenoprotein P and selenium in serum of elderly subjects in relation to other biomarkers of nutritional status and food intake. *J. Nutr. Biochem.* **1997**, *8*, 508–517.
48. Ravn-Haren, G.; Bugel, S.; Krath, B.N.; Hoac, T.; Stagsted, J.; Jørgensen, K.; Bresson, J.R.;
    Larsen, E.H.; Dragsted, L.O. A short-term intervention trial with selenate, selenium-enriched yeast and selenium-enriched milk: Effects on oxidative defence regulation. *Br. J. Nutr.* **2008**, *99*,
    883–892.
49. Alfthan, G.; Xu, G.L.; Tan, W.H.; Aro, A.; Wu, J.; Yang, Y.X.; Liang, W.S.; Xue, W.L.;
    Kong, L.H. Selenium supplementation of children in a selenium-deficient area in China: Blood selenium levels and glutathione peroxidase activities. *Biol. Trace Elem. Res.* **2000**, *73*, 113–125.
50. Bellisola, G.; Perona, G.; Galassini, S.; Moschini, G.; Guidi, G.C. Plasma selenium and glutathione peroxidase activities in individuals living in the Veneto region of Italy. *J. Trace Elem. Electrolytes Health Dis.* **1993**, *7*, 242–244.
51. Goldson, A.J.; Fairweather-Tait, S.J.; Armah, C.N.; Bao, Y.; Broadley, M.R.; Dainty, J.R.; Furniss, C.; Hart, D.J.; Teucher, B.; Hurst, R. Effects of selenium supplementation on selenoprotein gene expression and response to influenza vaccine challenge: A randomised controlled trial. *PLoS One* **2011**, *6*, e14771.
52. Ravn-Haren, G.; Krath, B.N.; Overvad, K.; Cold, S.; Moesgaard, S.; Larsen, E.H.; Dragsted, L.O. Effect of long-term selenium yeast intervention on activity and gene expression of antioxidant and xenobiotic metabolising enzymes in healthy elderly volunteers from the Danish Prevention of Cancer by Intervention by Selenium (PRECISE) pilot study. *Br. J. Nutr.* **2008**, *99*, 1190–1198.
53. Combs, G.F.; Jackson, M.I.; Watts, J.C.; Johnson, L.K.; Zeng, H.; Idso, J.; Schomburg, L.;
    Hoeg, A.; Hoefig, C.S.; Chiang, E.C.; *et al.* Differential responses to selenomethionine supplementation by sex and genotype in healthy adults. *Br. J. Nutr.* **2012**, *107*, 1514-1525.
54. Combs, G.F., Jr.; Watts, J.C.; Jackson, M.I.; Johnson, L.K.; Zeng, H.; Scheett, A.J.; Uthus, E.O.; Schomburg, L.; Hoeg, A.; Hoefig, C.S.; *et al.* Determinants of selenium status in healthy adults. *Nutr. J.* **2011**, *10*, 75.
55. Xia, Y.; Hill, K.E.; Byrne, D.W.; Xu, J.; Burk, R.F. Effectiveness of selenium supplements in
    a low-selenium area of China. *Am. J. Clin. Nutr.* **2005**, *81*, 829–834.
56. Burk, R.F.; Norsworthy, B.K.; Hill, K.E.; Motley, A.K.; Byrne, D.W. Effects of chemical form of selenium on plasma biomarkers in a high-dose human supplementation trial. *Cancer Epidemiol. Biomark. Prev.* **2006**, *15*, 804–810.
57. Alfthan, G.; Aro, A.; Arvilommi, H.; Huttunen, J.K. Selenium metabolism and platelet glutathione peroxidase activity in healthy Finnish men: Effects of selenium yeast, selenite, and selenate. *Am. J. Clin. Nutr.* **1991**, *53*, 120–125.

© 2014 by the authors; licensee MDPI, Basel, Switzerland. This article is an open access article distributed under the terms and conditions of the Creative Commons Attribution license (http://creativecommons.org/licenses/by/3.0/).
